# Association between percentage of smokers and prevalence of smoking attributable morbidity in Indonesia: one decade after implementation of smoke-free area regulation

**DOI:** 10.1186/s12889-022-14435-8

**Published:** 2022-11-28

**Authors:** Santi Martini, Kurnia Dwi Artanti, Arief Hargono, Sri Widati, Abdillah Ahsan, Yayi Suryo Prabandari

**Affiliations:** 1 Department of Epidemiology, Biostatistics, Population Studies and Health Promotion, Universitas Airlangga, Surabaya, Indonesia; 2 Faculty of Economics and Business, Universitas Indonesia, Jakarta, Indonesia; 3 Faculty of Medicine, Public Health and Nursing, Gadjah Mada University, Yogyakarta, Indonesia

**Keywords:** Smoking, Public health, Hypertension, Diabetes mellitus, Tuberculosis, Mental health

## Abstract

**Background:**

For more than ten years, Indonesia has health law, one of which states that local governments are mandated to establish Smoke Free Area (SFA). The results of 2018 National Basic Health Research shows tobacco consumption is still quite high and increasing compared to the results of 2007 and 2013 National Basic Health Research. The burden of disease in Indonesia is increasing every year.

**Methods:**

This study aims to describe SFA regulation and analyze the relationship between the percentage of smokers and the prevalence of smoking attributable morbidity. Data from the 2018 Basic Health Research in Indonesia with the number of units of analysis were 514 districts and cities level. The design of the study was cross-sectional study. The variables analyzed were the percentage of smokers, the prevalence of diabetes, hypertension, upper respiratory tract infections (URTI), pneumonia, lung tuberculosis, asthma, and mental emotional disorders. Geographical mapping of the distribution of District/City with Smoking-Free Areas was made using QGIS 3·16.

**Results:**

Around 72% of districts/cities in Indonesia already had local regulations of SFA after more than ten years implementation of the regulation of the health law. There was a significant relationship between the high percentage of smokers and the high prevalence of diabetes (*p* value: 0·000, PR: 1·342, 95%CI 1·135 to 1·587), hypertension (*p* value: 0·000, PR 1·631, 95%CI 1·252 to 2·124), and lung tuberculosis (*p* value: 0·008, PR 1·219, 95%CI 1·049 to 1·417) at the District/City level. However, there was no significant association between URTI, pneumonia, asthma, and mental emotional disorders.

**Conclusion:**

The percentage of smokers in an area was associated with diabetes, hypertension, and lung tuberculosis. The implementation of Smoke Free Area should be evaluated.

## Introduction

The proportion of the population aged 15 years who smoked and chewed tobacco in 2007 to 2013 in Indonesia tended to increase based on the 2007 National Basic Health Research by 34·2% and the 2010 National Basic Health Research by 34·7% [1, 2]. While at National Basic Health Research 2013, the proportion of active smokers every day at the age of ten years and over was 33·4% which then decreased in 2018 to 24·3%. Nevertheless, conditions in Indonesia at this time indicate that tobacco consumption is still quite high. There are 60·8 million adult men and 3·7 million adult women who are smokers. The 2018 Basic Health Research showed that 62·9% of men and 4·8% of women aged 15 years and over were tobacco users [3]. In addition, the data also show an increasing trend of tobacco use among children and adolescents. The prevalence of smoking in the 10–19 years age group has increased from 7·2% in 2013 to 9·1% in 2018 or almost 20% higher than the prevalence in the previous five years.

Trends in the number of non-communicable diseases cause changes in the burden of disease in Indonesia. Cases of catastrophic disease or diseases that require special expertise and therapy in handling, using sophisticated medical devices and/or requiring lifelong health services continue to increase [4]. It can be noticed from the absorption of health fund claims from the treatment of catastrophic diseases which is high in Indonesia. Each year, about 17–19% of the total cost of health care is for catastrophic diseases. The results of the National Health Insurance of Indonesia (BPJS Kesehatan) report on the use of catastrophic disease funds show that the total cost reached IDR 55·41 trillion or 18·58% of the total cost of health services in 2018 to 2020 [5]. The catastrophic disease with the highest cases and costs was heart disease with 13.041.463 cases and a cost of 10·2 trillion, in the second rank was cancer with 2.452.749 cases and cost of 3·5 trillion, and stroke in the third position with 2.127.609 cases and a cost of 2·5 trillion [6]. Many risk factors related to catastrophic disease, but the main cause is unhealthy lifestyle. Most of these catastrophic diseases are included in the list of diseases related to smoking called Smoking-Attributable Morbidity (SAM). The Centers for Disease Control and Prevention (CDC) regularly publish estimates of smoking-related mortality and the economic costs but the burden of smoking-related disease in the population has been less studied [7]. Estimates of smoking-attributable morbidity in the United States in 2000 found that 8·6 million people had 12·7 million smoking-attributable morbidity [8]. Diseases that are mostly the cause are chronic bronchitis and emphysema or are often classified as chronic obstructive lung disease (COPD).

The health and economic impacts of tobacco consumption outweigh the overall contribution of the tobacco business. Data from the Indonesian Ministry of Health estimates that the total direct and indirect costs of smoking reached almost IDR 440 trillion (USD 34 billion) in 2015. Then, if added to the impact of exposure to secondhand smoke and the lost opportunity cost of spending on tobacco i.e. spending that can be used to buy other commodities such as essential food, is much higher compare to the overall of the tobacco business. In addition, tobacco use also has a significant impact on public health because it can lead to the emergence of various chronic diseases in the productive age, which in turn causes high morbidity and premature mortality. Based on data from the World Health Organization [9], tobacco use in Indonesia is estimated to be the biggest cause of death for smokers, with around 225.700 people dying prematurely or about 15% of all deaths.

Indonesia has several rules controlling tobacco such as Tobacco Advertising, Promotion, and Sponsorship (TAPS) ban and pictorial health warnings on the tobacco packaging and labeling. Another effort of the Indonesian government in preventing and overcoming the adverse effects of cigarette smoke is the application of Smoke Free Area (SFA) in accordance with Law Number 36 of 2009 concerning Health which requires Local Governments to establish a Smoke Free Area. SFA is a room or area that is prohibited for production, sales, advertising, promotion or smoking. Smoke Free Area here in after abbreviated as SFA, is a room or area that is declared prohibited for smoking or producing, selling, advertising, and/or promoting tobacco products. Based on these regulations, the non-smoking areas include health service facilities; place of teaching and learning process; where children play; worship place; public transportation; workplace; and public places and other designated places. The SFA designated as an effort to protect the community against the risk of health problems resulted of the polluted environment by cigarette smoke. The regional governments in Indonesia are obliged to establish a smoke-free area in each of its territory. As a policy concession, specifically for workplaces, public places, and other places, it may provide a certain place for smoking, which is commonly called a smoking room. Any person who intentionally violates the SFA as referred to the law, shall be subject to a maximum fine of Rp. 50,000,000.00 (fifty million rupiah). Tobacco control regulations in various countries have succeeded in protecting non-smokers, increasing smoking cessation, and reducing cigarette consumption [10]. Since this law was passed in 2009, various regions in Indonesia have begun to make regulations regarding SFA in their respective regions, but until now this rule has not been 100% implemented in all regions. In 2012 out of 497 regencies/cities in Indonesia, only 22 regencies/cities had local regulations on SFA, and then in 2014 the number of regencies/cities that had local regulations on SFA increased to 49 spreads over 13 provinces (out of 34 provinces) in Indonesia [11].

Cigarettes are still a problem in Indonesia with a high number of consumers. Smoke Free Areas as an effective form of protection from the dangers of cigarette smoke, provide a clean and healthy environment, and expected to control smoking behavior which can affect the number of smokers in each area. Cigarette taxes provide support to local governments to achieve better health services. This study aims to describe the Smoke Free Area regulation and analyze the relationship between the percentage of smokers and the prevalence of smoking attributable morbidity.

## Method

This study uses a quantitative method with a cross-sectional design. Secondary data was obtained from the 2018 Basic Health Research national survey data. Basic Health Research is a 5-year survey conducted by the Ministry of Health of the Republic of Indonesia that describes information on health status and information on the magnitude of the problem of risk factors related to health status, as a consideration in formulating health development policies in Indonesia. Until this manuscript was compiled, the latest Basic Health Research and the data officially published is in 2018. The unit of analysis used in this study was 514 districts/cities in Indonesia. The variables studied were the percentage of smokers and outcome of this study were the prevalence of smoking-attributable morbidity, namely diabetes, hypertension, upper respiratory tract infections (URTI), pneumonia, asthma, lung tuberculosis, and mental emotional disorders. The measurement result category of these variables is divided to be high if the percentage or prevalence of smokers and smoking-related diseases is above the national figure. The percentage of smoking is categorized as high if the percentage of smokers is above national percentage, was > 24·5% for the 2018 National Basic Health Research. While the diseases prevalence was categorized as high if the prevalence of diabetes was > 1·5%; hypertension was > 8·36%; URTI was > 4·4%; pneumonia was > 2·0%; lung tuberculosis was > 0·42%; asthma was > 2·4%; and mental emotional disorders was > 9·8%. On the other hand, it is said to be low if the percentage of smokers and the prevalence of smoking-attributable morbidity are below the national figures. Data analysis using Chi-Square Test and Prevalence Ratio.

Smoker variable is population with smoking habit every day in the last one month which was asked to respondents with age more than ten years. The diseases variable was asked to respondents aged > 15 years. Diagnosed of diabetes mellitus based on American Diabetic Association criteria, hypertension based on Joint National Committee (JNC) VII, asthma based on medical doctor examination, lung tuberculosis based on thorax photo or sputum examination or both, pneumonia based on three questions (thorax photo, medical doctor examination and, symptoms), URTI based on two questions (medical doctor examination and symptoms), and mental emotional disorders based on 20 questions of Self-Reporting Questionnaire (SRQ). All of those data could be accessed on the official website https://www.litbang.kemkes.go.id/laporan-riset-kesehatan-dasar-riskesdas//. Furthermore, the mapping was made using the QGIS 3·16 application and District/City data with the 2021 SFA regulation sourced from data from the Indonesian Health Service Association (ADINKES) [12]. The Indonesian Health Service Association (ADINKES) is an association organization established to meet the aspirations and participation of Health Offices throughout Indonesia. ADINKES has a vision to become the main partner of the Government and Regional Governments throughout Indonesia in realizing the Health Service and its Technical Implementation Units as a trusted and reliable health affairs implementer.

## Results

**Fig. 1:**
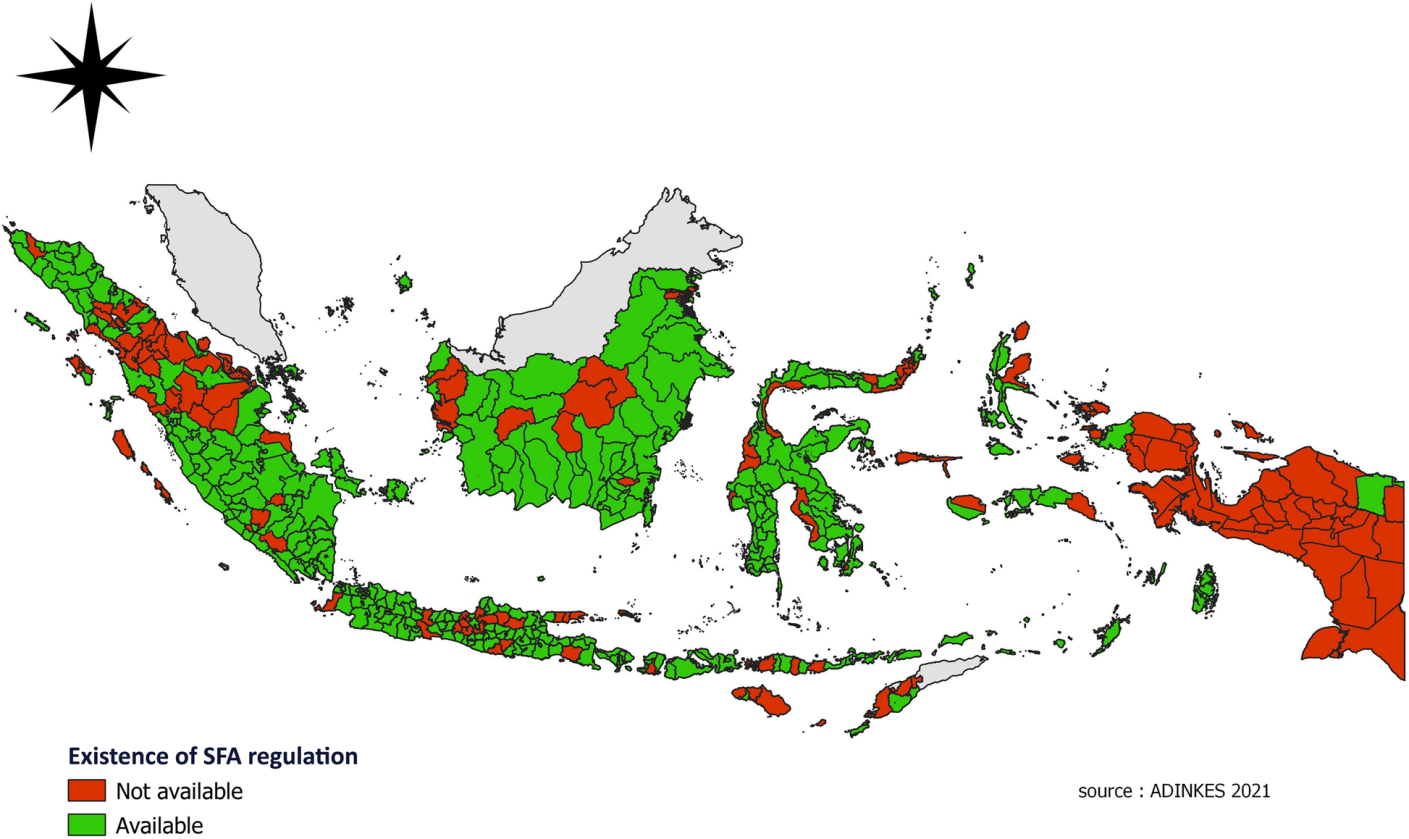
The distribution of SFA regulations at the District/City level in Indonesia in 2021

In 2021, out of 514 districts/cities in Indonesia, there are 369 districts/cities (71·8%) that already had SFA regulations in their regions and as many as 145 districts/cities (28·2%) did not have regulations on SFA in the area (Fig. 1). The number was much higher than the number of the districts/cities with SFA regulation before 2009. These regulations can be in the form of local regulations; regent’s or mayor’s regulations; or decrees. Local regulations were formed based on an agreement between the government (executive role) and parliament (legislative role). While other regulations are made only by the government (executive role) without involving parliament.

**Fig. 2:**
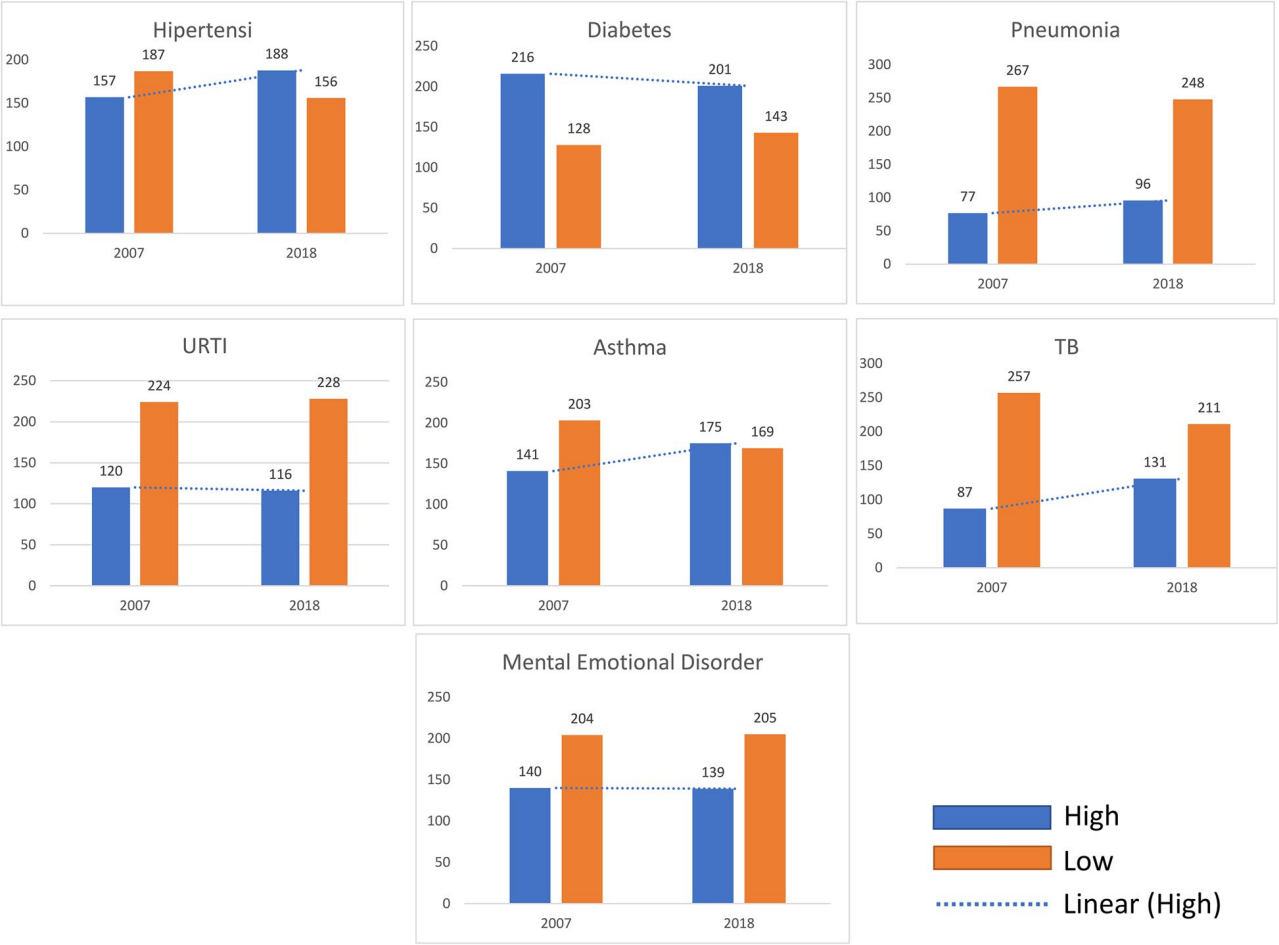
Trendline of the prevalence of Smoke Attributable Morbidity from 2007 to 2018

The condition of the prevalence of Smoke Attributable Morbidity from 2007 to 2018 shows the difference between before the regional regulation on Non-Smoking Areas in 2009 and the allocation of cigarette taxes for the health sector was implemented, with the condition after the enactment of regional regulations on non-smoking areas that have been implemented by most regions in Indonesia, as well as affirmation of the allocation of cigarette taxes for the health sector (Fig. 2). The data is analyzed from 72% districts/cities that have SFA which the total is 344 districts/cities (districts/cities with expansion and missing data in 2007 are not included). The trendline of each Smoke Attributable Morbidity shows the number of districts/cities with hypertension, pneumonia, asthma, and TB in the high category has increased. Meanwhile, the number of districts/cities with diabetes, URTI and mental emotional disorders in the high category has decreased.

### Overview of Smoking Attributable Morbidity in Districts/Cities of Indonesia

**Table 1:** Description of Districts/Cities with percentage of Smokers and Prevalence of Smoking Attributable Morbidity in Indonesia

Variable	District and City (*n* = 514)		Missing data
	**High (%)**	**Low (%)**	
Smoking	217 (43·8)	279 (56·3)	18
Diabetes Mellitus	322 (64·5)	173 (35·5)	1
Hypertension	218 (44·6)	278 (55·4)	-
URTI	184 (36)	312 (64)	-
Pneumonia	151 (30·4)	345 (69·6)	-
Lung TBC	196 (45·3)	298 (54·7)	-
Asthma	222 (39·5)	274 (60·5)	2
Mental emotional disorder	203 (41·2)	293 (58·8)	-

The percentage of smokers in districts or cities according to 2018 National Basic Health Research with a high category was 217 districts/cities (43·8%) after ten years implementation of Health Law. It showed that as many as 217 districts or cities in Indonesia had more than 24·3% of smokers. The number of districts/cities which had high prevalence of diabetes were 322 districts/cities (64·5%). It meant that 322 districts/cities had prevalence of diabetes more than 1·5% as the national figure. The number of districts/cities which had high prevalence of hypertension were 218 districts/cities (44·6%). It meant that 218 districts/cities had prevalence of hypertension more than 8·36% as the national figure. The number of districts/cities which had high prevalence of lung tuberculosis were 196 districts/cities (45·3%). It meant that 196 districts/cities had prevalence of lung tuberculosis more than 0·42% as the national figure. While the disease with the high category and the lowest number was pneumonia with 151 districts/cities (30·4%). It meant that 151 districts/cities had prevalence of pneumonia more than 2·0% as the national figure (Table 1).

### The Association between Smoking and Smoking-Attributable Morbidity in Districts/Cities of Indonesia

**Table 2:** Analysis between percentage of smokers and prevalence of smoking attributable morbidity in Indonesia based on Districts/Cities as unit of analysis

Variable	Number of Districts/Cities with High Percentage of Smokers		Missing data	p	PR	95% CI
	**Yes**	**No**				
Prevalence of Diabetes						
High	161	161	18	·000	1·342	1·135–1·587
Low	56	117				
Prevalence of Hypertension						
High	115	103	1	·000	1·631	1·252–2·124
Low	102	176				
Prevalence of URTI						
High	79	105	-	·779	·981	·856–1·123
Low	138	174				
Prevalence of Pneumonia						
High	76	75	-	·051	1·125	·997–1·270
Low	141	204				
Prevalence of Lung TBC						
High	100	96	-	·008	1·219	1·049–1·417
Low	116	100				
Prevalence of Asthma						
High	103	119	2	·285	1·092	·928- 1·284
Low	114	160				
Prevalence of Mental Emotional Disorder						
High	99	104	-	·061	1·153	·991–1·343
Low	118	175				

Result of the analysis of the association between the percentage of smokers and the prevalence of smoking-attributable morbidity showed significant results with *p* value < 0·005 on the variables of diabetes mellitus (*p* = 0·000), hypertension (*p* = 0·000), and lung tuberculosis (*p* = 0·008). Districts/Cities with a high percentage of the category had a risk of 1·342 times (CI; 1·135-1·587) to be districts/cities with a high prevalence of diabetes, 1·631 times (CI: 1·252-2·124) with high prevalence of hypertension, and 1·219 times (CI: 1·049 − 1·417) with high prevalence of lung tuberculosis as well. However, URTI, pneumonia, asthma, and mental emotional disorders were not associated significantly with the percentage of smokers. Although pneumonia (*p* = 0·051) and mental emotional disorders (*p* = 0·061) had p values that were close to significant value (Table 2).

## Discussion

Smoke Free Area regulation is one of the government’s efforts to control diseases caused by smoking and exposure to cigarette smoke in the environment. A meta-analysis research shows that smoke free area regulation as implementation of WHO’s recommended tobacco control policies (MPOWER) was associated with substantial benefits to health especially on perinatal and children health, especially who got in tobacco smoke exposure [13, 14]. The WHO MPOWER recommendation were able to reduce the adult daily smoking prevalence significantly the countries with higher initial tobacco control preparedness and higher smoking burden [15]. Children were among the most exposed to cigarette smoke or become passive smokers compared to other age groups. Other research show that smoking regulation associated with reduction in severe asthma exacerbations requiring hospital admission [16]. The Smoke Free Area regulation was expected to have an effect on reducing the number of smokers. This is in accordance with Rahajeng’s research in 2015 which stated that the application of SFA regulations and legislation can reduce the proportion of smokers every day. One of the areas, for example the Special Region of Yogyakarta, the number of smokers every day from 2007 to 2013 decreased by 2·6% [17]. Even though the regulation of Smoke Free Area had been implemented since 2009, the number of smokers in Indonesia was still high. It showed that there was low compliance on the regulation due to poor enforcement, as the study result of Ravara et al. (2013) [18]. This was also related to Smoking-Attributable Morbidity which was also still a large disease burden for the country.

The results showed that districts/cities with high smoking category had a high prevalence of diabetes mellitus, hypertension, and lung tuberculosis. The strong association between smoking and the diabetes is consistent with studies and the development of micro and macrovascular complications. Smoking causes changes in insulin secretion by pancreatic cells and can also cause insulin resistance associated with impaired glucose metabolism. In addition, smoking-induced endothelial dysfunction has a key role in the development of vascular complications in this condition [19]. Smokers had a 2·30 (95%CI 1·47 to 3·60) risk of developing diabetes mellitus than non-smokers [20]. In this study, districts/cities with high smoking category had a risk of 1·631 times (95%CI 1·252 to 2·124) to become districts/cities with high prevalence of diabetes mellitus.

The results showed that districts/cities with a high percentage of smokers had a high prevalence of hypertension as well (95%CI 1·252 to 2·124). Smoking was significantly correlated with higher blood pressure, especially among former smokers and new smokers [21]. Hypertension had been common and is still considered as one of the main risk factors for coronary heart disease. Endothelial dysfunction, increased arterial stiffness, and changes in platelet function caused by smoking exposure contribute to increased blood pressure, which is strongly associated with hypertension. The results of another study [22] also showed that cigarette smoke is a factor that can cause functional damage, especially to the endothelium due to the effects of nicotine and carbon monoxide.

Meanwhile, the high percentage of smokers in districts/cities also showed a significant relationship with a high prevalence of lung tuberculosis (95%CI 1·049 to 1·417). Previous studies have stated that excessive tobacco consumption in men (⩾20 cigarettes per day) was a risk factor for tuberculosis (OR: 4·509; CI: (1·971–10·859) [23]. However, Ghambir et.al’s study explains that the association between smoking and tuberculosis can be influenced by confounding factors such as socioeconomic conditions, population density at home, previous contact with tuberculosis patients, previous TB infection, malnutrition, use of intravenous drugs, alcohol consumption, and high-risk occupations [24].

The high blood glucose associated with diabetes can damage the blood vessels and nerves that control the heart. Over time, this damage can lead to heart disease. Heart disease is associated with various complications of other diseases, from stroke to kidney failure [25, 26]. Complications of diseases such as these are more likely to cause disability and loss of productivity, especially if they occur in people of productive age. Meanwhile, the total health care expenditure due to Smoking-Attributable diseases is 5·7% of global health expenditure or reaches US$422 billion if calculated according to Purchasing Power Parity (PPP) in 2012 [27].

There were 706 million DALYs (Disability Adjusted Life Year) worldwide attributable to non-communicable diseases in 2017 and diabetes is one of the top five causes of DALYs by association with major risk [28]. The Global Burden of Diseases, Injuries, and Risk Factors Study (GBD) 2017 showed that stroke was the third leading cause of death and disability combined (measured by disability-adjusted years of life [DALYs]). Meanwhile in Indonesia, the main causes of DALYs in 2016 were ischemic heart disease, cerebrovascular disease, diabetes, and lung tuberculosis which took the fourth position. Diabetes recorded an average rate from 2006 to 2016 of 54·9%, after previously in 1990–2006 it was 81·6% [29].

Many areas with SFA regulations are not followed by a good public health status as well. This can be seen from the high percentage of smokers and the high prevalence of smoking-related diseases. So, it’s time not only to add regions that have SFA regulations but also to apply the regulations correctly, which will be effective in reducing smokers and the diseases they cause. Although in this study asthma was not associated significantly with smokers, as we know there were many studies showed that there are association between cigarette smoke exposure and asthma, as the result showed that smoking from younger age is a dominant factor in the incidence of Chronic Obstructive Pulmonary Disease, such as asthma [30, 31].

The data in this research is secondary data which based on 2018 Basic Health Research, the smoking variable was defined as anyone smoking every day in the last month which was asked to respondents above 10 years old, however, the disease’s variable was asked to respondents aged > 15 years, this is the limitation of the study. In addition, the number of smokers aged 10–15 is 0.7%. The prevalence data of smoking-related morbidity/diseases consist of diseases which age manifestations mostly on people aged > 30 years, therefore, we may consider this will not give much differences affect the analysis.

## Conclusion

This study showed that around 72% of districts/cities in Indonesia already have local regulations of Smoke Free Area. There was a significant association between the high percentage of smokers with high prevalence of smoking attributable morbidity, such as diabetes mellitus, hypertension, and lung tuberculosis in the District/City in Indonesia. Certainly, Smoke Free Area should be evaluated in the implementation. It will have an impact on the health status of the people in the area at this time and in the future considering that the disease is a chronic disease and requires very high treatment costs.

## Data Availability

All data set generated and analysis are available in the article. The raw data is available in the website https://www.litbang.kemkes.go.id/laporan-riset-kesehatan-dasar-riskesdas//.
